# Impaired Visual Motor Coordination in Obese Adults

**DOI:** 10.1155/2016/6178575

**Published:** 2016-11-22

**Authors:** David Gaul, Arimin Mat, Donal O'Shea, Johann Issartel

**Affiliations:** ^1^School of Health and Human Performance, Dublin City University, Dublin, Ireland; ^2^Weight Management Service, St. Columcille's Hospital, Loughlinstown, Ireland; ^3^Department of Endocrinology, St. Vincent's University Hospital, Dublin 4, Ireland

## Abstract

*Objective.* To investigate whether obesity alters the sensory motor integration process and movement outcome during a visual rhythmic coordination task.* Methods.* 88 participants (44 obese and 44 matched control) sat on a chair equipped with a wrist pendulum oscillating in the sagittal plane. The task was to swing the pendulum in synchrony with a moving visual stimulus displayed on a screen.* Results.* Obese participants demonstrated significantly (*p* < 0.01) higher values for continuous relative phase (CRP) indicating poorer level of coordination, increased movement variability (*p* < 0.05), and a larger amplitude (*p* < 0.05) than their healthy weight counterparts.* Conclusion.* These results highlight the existence of visual sensory integration deficiencies for obese participants. The obese group have greater difficulty in synchronizing their movement with a visual stimulus. Considering that visual motor coordination is an essential component of many activities of daily living, any impairment could significantly affect quality of life.

## 1. Introduction

According to World Health Organization (WHO) figures from 2014, 39% of adults (1.9 billion people) were overweight with more than 600 million of these being found to be obese. This is particularly alarming considering the worldwide prevalence of obesity has doubled since 1980. The obesity problem also seems set to continue with 42 million children under the age of 5 being either overweight or obese in 2013 [1]. Obesity is the major health concern of this generation. Obesity has been linked to increased risk of other diseases such as stroke, cancer, cardiovascular disease, obstructive sleep apnea (OSA), type II diabetes mellitus (T2DM), hypertension, and mental health problems [2]. In addition obesity has also been found to be associated with increased risk of fall, reduced quality of life, and problems with activities of daily living [3–5]. As such, many obese individuals report how clumsiness has affected their daily lives [6]. In addition to this, subjectively health care practitioners frequently report obese patients as being clumsy or awkward in their performance of fine motor skill activities such as signing forms or tying laces. The increased mechanical constraints placed on individuals as a result of increased adiposity reduce balance and increase risk of falls [3, 7] in addition to reducing physical activity [8]. However a number of studies exist suggesting that differences found in the balance and fine motor skill proficiency between obese individuals and normal weight peers might have a neuromuscular component as opposed to only being caused by mechanical impairment as traditionally suggested [9–14]. A study by D'Hondt et al. (2008) investigated balance and postural sway between obese and normal weight children. In this study, children carried out using a traditional 9-hole peg task activity while in altered postural conditions, standing on a balance beam or while being seated. As expected, obese children scored worse for the balance beam activity most likely due to the increased demands placed on their postural control due to their excess mass. However, more surprisingly, obese children were also found to score significantly worse than their normal weight peers on the task while in the seated position. In addition to this, Gentier and colleagues (2013) found that obese children demonstrate impaired fine motor skills in addition to gross motor skills when compared to their normal weight peers. As fine motor skills are not directly affected by excess mass, this would suggest that other factors exist to impede obese individual's motor control [13]. This leads to the suggestion that the problem may lie in the sensory integration process [9, 10, 12, 13, 15].

The sensory integration process relies on the complex interaction between the individual, the task being carried out, and the environment in which it takes place [16]. This relationship between the sensory integration process and motor behaviour has been extensively studied in typically developing adults [17, 18] and more recently to gain a greater understanding of how this process is altered in autistic [19] and schizophrenic [20] individuals. These pendulum based paradigms allow for the environment, task, and individual constraints to be maintained which allows the mechanisms underlying this complex behaviour to be examined. An individuals motor behaviour is the observable output from the complex interaction between all components of the system [21]. As an individual's interaction with the environment requires movement, any difficulties with how they control their motor output could have large consequences on their everyday life increasing the complexity, difficulty, and attentional costs associated with any motor task and the potential for errors.

Recent research has begun to find association between cognitive performance and obesity [22, 23]. Reviews by Smith et al. (2011), Wang et al. 2016, Liang et al. (2014), and Prickett et al. (2015) have found obesity to be associated with cognitive performance over the course of the lifespan. These links between cognitive performance and obesity further strengthen the rationale that obesity may have a neural component. Obesity is also associated impaired motor control across the lifespan which impacts upon activities of daily living and health [24–26]. Studies in children have shown obese or overweight children perform worse than their normal weight peers in both fine and gross motor skills [12, 13, 27]. The argument for poor motor coordination potentially being a predictor of future obesity suggested by Gentier et al. (2013) and D'Hondt et al. (2008) is strengthened by two longitudinal studies [28, 29]. The first was carried out by Osika and Montgomery (2008) that found that teachers assessment of poor coordination at age 7 and standardized motor coordination tests at age 11 predicted obesity at age 33 even when correcting for a range of factors such as gender and social class. The second was reported by Chandola et al. (2006) that found that lower IQ scores in childhood were associated with obesity and weight gain in adulthood. These findings suggest that should children underperform at a young age, this could lead to detrimental effects persisting throughout adulthood.

In adults, a number of early studies that have examined the relationship between cognitive performance and obesity failed to control for factors such as diabetes, cholesterol, and smoking [30, 31]. Since then several studies have found negative relationships between BMI and multiple cognitive domains in adults such as attention, memory, numeracy, executive function, and motor control [24, 32]. A number of studies have also used imaging techniques to find a negative association between BMI and brain activity and structure suggesting decline in cognitive performance [33, 34]. A study that investigated the relationship between obesity and cognitive function on the participants of the Framingham heart study found adverse effects on cognitive function in obese participants and suggested that earlier onset and long term obesity could adversely affect later cognitive performance [35]. In adults, research has found increased BMI and BP to be associated with impaired manual dexterity and reduced motor speed [36]. In addition to this, a study by Fedor and Gunstad (2013) examined the cognitive function in high level collegiate athletes, some of whose BMI were overweight or obese. Interestingly, it was found that higher BMI was associated with reduced cognitive function, even in the sample expected to have good cardiovascular fitness levels [23]. Of more interest is that visual motor speed was one of the composites of cognitive function that was found to be significantly negatively associated with higher BMI. This would further support the rationale that obesity influences the sensory integration process rather than solely being a mechanical constraint.

This study aims to specifically investigate the sensory integration process during a visual motor task and whether obesity interacts with it. This study used visual cueing as vision is the dominant sensory modality during many daily living activities [37]. As such, this allowed us to investigate the potential impact of visual motor coordination difficulties in everyday life of obese individuals. To do so, this study utilised a pendulum based paradigm as it has been extensively used in motor control research over the past 20 years to ensure reproducibility [37, 38]. This allowed us to compare performance output between studies assessing normal participants and participants with motor difficulties such as schizophrenia and autism [19, 20]. We hypothesize that obese individuals coordination during a visual rhythmic motor task would be inferior compared to their normal weighted peers. This would lead to the potential for a neuromuscular component previously not accounted for in obesity research currently.

## 2. Methods

### 2.1. Subjects

Forty-four right-handed obese patients and 44 age- and gender-matched controls participated in this study. The obese participants attended the Weight Management Service, an outpatient-based lifestyle-intervention weight management program designed to promote weight management through dietary changes and increased physical activity in St. Columcilles Hospital, Loughlinstown, Co Dublin, Ireland. All patients had a BMI > 40.0 in order to be admitted into the program. Prior to recruitment all participants were screened for any comorbid condition or medication deemed to potentially influence performance. As such patients suffering from diabetic retinopathy and poor vision or had laser eye surgery, osteoarthritis, injury/chronic pain of the right arm, untreated hypothyroidism, stroke, and peripheral neuropathy or receiving antipsychotic medication were excluded from the study. A number of participants had OSA (*N* = 18) and T2DM (*N* = 13). All participants were also screened for colour blindness using the Ishihara Test of Colour Blindness short form. All participants had their height, weight, and date of birth recorded prior to the assessment. Height was measured to the nearest 0.1 cm while standing barefoot using a portable stadiometer (Leicester Height Measure). Body weight was measured using a mechanical weighing scale (Seca Mechanical Weight Scales Model 761) and corrected to the nearest 0.5 kg (Table 1).

### 2.2. Procedure

**Figure 1:**
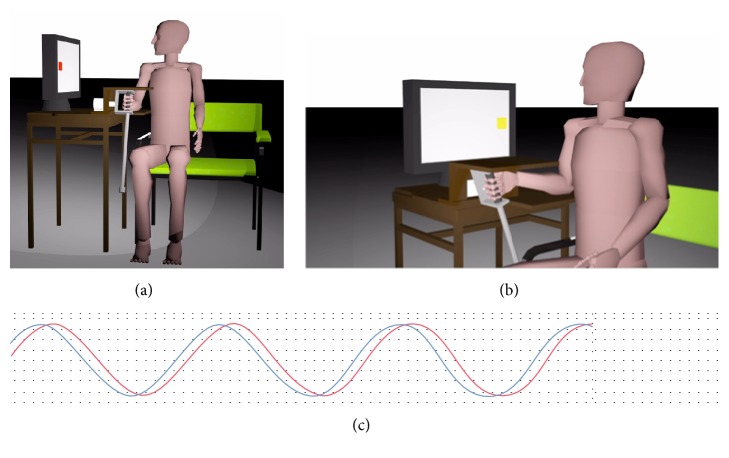
Experimental set-up. Participants sat in a bariatric chair and were asked to swing the pendulum forward as the square moved left and faded to a red colour (a) and backward as the square moved right on the screen fading to a yellow colour (b) synchronizing the endpoint of the movements with the square's endpoints. From the resting position of the pendulum, participants swung the pendulum to a 45° angle to the left and a 45° angle to the right. (c) Participants' pendulum position was recorded using a potentiometer and compared with the computer generated stimulus (cf. Procedure).

This task involved a visual motor coordination task where the participant synchronized his movement with computer generated stimuli using a hand-held pendulum [17]. Participants sat in a bariatric chair and placed their right arm in a forearm support. The visual stimuli were presented on a screen (Dell Trinitron UltraScan 1600HS Series CRT Monitor, Model D1626HT) placed 1 meter from the participant at eye level. The visual stimuli consisted of a square (5.2 cm × 5.2 cm) that faded from red to yellow while oscillating horizontally across the screen on a grey background in a sinusoidal manner with amplitude of 28 cm. The experiment was controlled and run through Matlab using a Graphical User Interface as part of a Psychophysics Toolbox Extension [39]. Participants were asked to swing the pendulum forward as the square moved left and backward as the square moved right on the screen synchronizing the endpoint of the movements with the square's endpoints (Figure 1). Participants were prevented from viewing the pendulum's movements and their forearm by a wooden cover and a cloth curtain.

The experiment consisted of three phases: (1) preferred frequency calculation, (2) familiarisation, and (3) experimentation. For the preferred frequency calculation participants were instructed to swing the pendulum in a dark room for two minutes at a pace that was “most comfortable” for them which they could swing at “all day long" [38]. From this, the frequencies for the −20% and +20% conditions were calculated. During familiarisation, each subject carried out one practice trial for each of the 3 experimental conditions. The participants received additional presentations of the stimuli if required to ensure understanding of the different experimental conditions.

### 2.3. Experimentation

Following familiarisation phase, subjects carried out 2 blocks of experimentation. One block of the experiment consisted of 3 frequencies (preferred, +20%, and −20%) being played in a randomised order. Participants completed one trial of each of the 3 randomised conditions for each of the 2 blocks. There was a 30-second break after each 40-second trial and a 2-minute break between blocks to prevent fatigue.

### 2.4. Data Reduction

All data was recorded at 100 Hz using a Measurement Computing Data Acquisition Device (measurement computing USB-1608FS) for analysis. The degree of coordination between the participant and the stimulus was assessed using continuous relative phase (CRP) (see Figure 1(c)). CRP was calculated using a Hilbert Transform and scaled between 0° (indicating perfect synchrony) and 180° (complete opposite). These two stable states are referred to as in phase or anti phase. For this type of task, it is important to note that that participants' coordination naturally is attracted to either of these states. The first 10 seconds and last cycle of each trial were removed in order to eliminate distortions caused by Hilbert Transform on the computation. The variability of coordination was assessed using the standard deviation (SD) of CRP calculated from the CRP values. Participants movement amplitude for each trial was also measured. All data was averaged across each of the trials for the 3 experimental conditions.

### 2.5. Statistical Analysis

All statistical analysis was performed using SPSS (IBM SPSS Statistics 19). Independent samples *t*-tests were carried out to investigate any potential influence of OSA and T2DM on performance. A 3 × 2 × 2 repeated measures ANOVA on CRP, SD CRP, amplitude, and CRP timing was carried out to examine the influence of frequency, weight group and gender on visual motor coordination. Sphericity was assessed and the Greenhouse and Geisser correction for degrees of freedom were applied when sphericity was not met. Post hoc analysis using the Bonferroni correction was carried out.

## 3. Results

Independent samples *t*-tests revealed no significant difference between subject's effects for OSA and T2DM on each of the variables used.

### 3.1. Continuous Relative Phase (CRP)

There was no significant main effect for frequency *F*(1.84,154.58) = 3.12, *p* > 0.05. There was a significant weight group × gender interaction effect *F*(1,84) = 4.02, *p* < 0.05. Post hoc tests revealed that CRP scores for the male and female obese group (52.06 and 32.25, 95% CI [42.35, 61.76], [23.39, 41.12], resp.) differed significantly from those of the control group (15.79 and 14.72, 95% CI [6.08,25.50], [5.86,23.58]), respectively.

### 3.2. Standard Deviation of CRP

The gender × frequency × weight group ANOVA on SD CRP did not reveal any significant interaction effects. There was a significant main effect found for group *F*(1,84) = 283.58, *p* < 0.01 with OB group (M = 22.16 SD = 14.04, 95% CI [19.63,25.10]) demonstrating significantly more variable coordination compared to the NW group (M = 10.36, SD = 4.83, 95% CI [7.67,13.14]). There was a significant main effect for frequency *F*(1.8,151.28) = 3.38, *p* < 0.05. Post hoc tests revealed that participants had significantly more variable coordination, *F*(1,84) = 4.21, *p* < 0.05 for the −20% condition (M = 16.64, SD = 11.63, 95% CI [14.54,18.83]) compared to preferred frequency condition (M = 14.82, SD = 11.91, 95% CI [12.75,17.16]). There was no significant main effect found for gender (*F*(1,84) = 1.89, *p* > 0.05).

### 3.3. Amplitude

There were no significant interaction effects in gender, frequency, or weight group found for participant's amplitude. There was a main effect found for weight group *F*(1,84) = 10.69 *p* < 0.01 with the obese group being found to swing the pendulum with 24% greater amplitude (M = 61.04, SD = 17.57, 95% CI [55.34,65.50]) compared to the control group (M = 49.29, SD = 20.40, 95% CI [43.53,53.68]). There was also a significant main effect for gender, *F*(1,84) = 15.71 *p* < 0.01 with females swinging the pendulum over a 30% greater amplitude (M = 61.68, SD = 18.41, 95% CI [56.83,66.52]) compared to male participants (M = 47.36, SD = 18.77, 95% CI [42.05,52.66]). There was a significant main effect found for frequency, *F*(1.75,146.61) = 11.42, *p* < 0.01. Contrasts carried out revealed that participants swung the pendulum at a significantly greater amplitude, *F*(1,84) = 25.40 *p* < 0.01, for −20% conditions (M = 52.29, SD = 18.58, 95% CI [48.11,55.44]) compared to preferred speed conditions (M = 57.48, SD = 20.89, 95% CI [52.83,60.66]).

### 3.4. Standard Deviation of Amplitude

There were no significant interaction effects in gender, frequency, or weight group found for SD of participant's amplitude. There was no significant effect found for frequency (*F*(1.83,153.36) = 1.69, *p* > 0.05). There was a significant main effect for weight group *F*(1,84) = 19.25, *p* < 0.01 with obese participants demonstrating higher variability in their amplitude (M = 6.93, SD = 3.88, 95% CI [6.11,7.61]) when compared to normal weight controls (M = 4.57, SD = 2.73, 95% CI [3.76,5.26]). There was also a significant main effect found for gender *F*(1,84) = 6.79, *p* < 0.05. Further investigation found that female participants demonstrated more variable amplitude (M = 6.38, SD = 3.76, 95% CI [5.67,7.10]) compared to males (M = 4.99, SD = 3.15, 95% CI [4.20,5.77]).

## 4. Discussion

We found that obese participants demonstrated lower and more variable coordination levels with greater amplitude of movements than their normal weight peers. As this experimental paradigm controls the mechanical and environmental factors that frequently influence motor behaviours of individuals, this leads us to question the source of these differences. One potential hypothesis is that that these differences result from problems with the underlying perception and integration of sensory information that govern the movement process.

**Figure 2:**
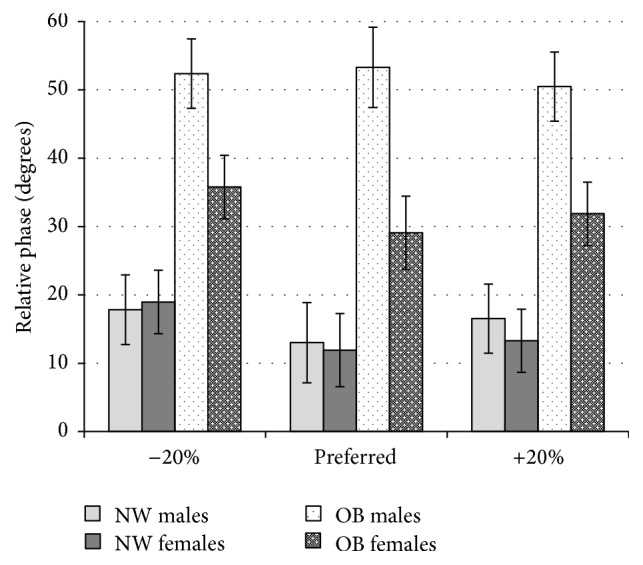
Mean continuous relative phase (CRP) values shown for normal weight (NW) and obese (OB) participants divided by gender for all 3 frequency conditions (−20%, preferred frequency, and +20%).

**Figure 3:**
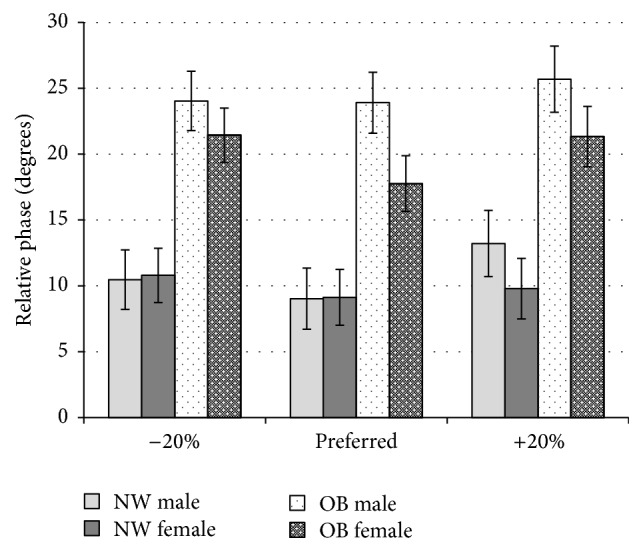
Mean standard deviation values shown for normal weight (NW), and obese (OB) participants by gender for each frequency (−20%, preferred frequency, and +20%).

The values for CRP obtained for the obese group were significantly higher than the normal weight group whose performance coincides with values found in previous research [17]. This finding indicates that obese subjects had greater difficulty in synchronizing their movements and maintaining their synchrony with the stimulus (Figure 2). The obese group also demonstrated significantly higher values for SD CRP indicating more variable coordination (Figure 3). This unstable pattern of coordination is a demonstration of their need to constantly readjust their coordination with the stimulus. It is important to note that usual performance values, for pendulum task, are around 20° [38]. Hence, such task is considered as quite simple with performance-ceiling values reached after 2-3 familiarisation trials. We can then expect a direct transfer of this low performance level on their ability to coordinate movement during everyday life activities such as brushing ones hair or using utensils to eat directly affecting individual's quality of life. In addition, visuospatial coordination is a vital component of more complex forms of movement such as those involved in many forms of physical activity and sports participation or while driving a car. In addition to this, movement tasks that also require increased cognitive load such as decision-making would be an additional demand on the sensory integration process underlying movement and further increase the task difficulty for these individuals. This could prove to be an additional barrier to participation in many forms of physical activity.

**Figure 4:**
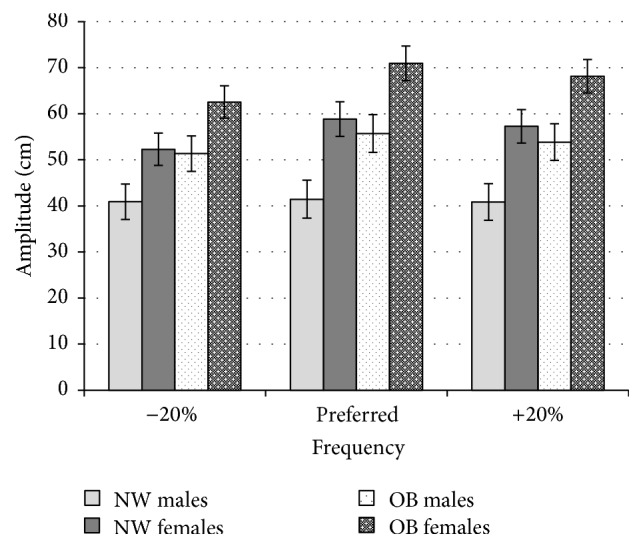
Mean amplitude values over which participants swung the pendulum shown for normal weight (NW) and obese (OB) participants divided by gender for all 3 frequency conditions (−20%, preferred frequency, and +20%).

**Figure 5:**
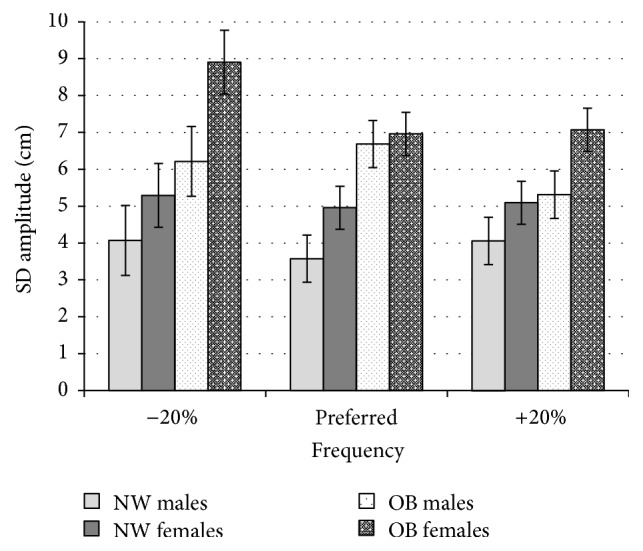
Mean standard deviation of amplitude shown for normal weight (NW), and obese (OB) participants divided by gender for all 3 frequency conditions (−20%, preferred frequency, and +20%). Greater values imply a greater degree of variability in the amplitude over which participant swung.

In terms of movement amplitude, the obese group also demonstrated greater (Figure 4) and more variable (Figure 5) amplitude of movement compared to their normal weight counterparts. In addition, the increased variability of the amplitude for the obese group also reveals the control of the pendulum swing is reduced. The lack of consistency in repetitive task reveals that the task difficulty for the obese group is higher in comparison with the normal weight group. The movement patterns stability is an indicator of the control a person has in continuous repetitive situations. The support of the forearm and natural frequency of the pendulum (which requires little force to drive) during the experiment could be seen to remove any biomechanical influence of musculature or mass of the arm. This finding suggests that obese individuals may employ a slightly different coordinative strategy to synchronize their movements with the stimulus compared to normal weight individuals. It could be the case that obese individuals alter the amplitude over which they swing to help maintain a similar angular velocity as a compensatory measure to aid synchrony with stimulus. This finding suggests that obese individuals employ different behavioural strategies in the coordination of movement to overcome barriers experienced during the task.

The significant effects found for frequency for measures of variability of coordination and amplitude of movement for both obese and normal weight groups are also in line with previous research on normal population [17]. As expected, the −20% condition is more difficult to synchronize when compared to other preferred frequencies or +20%. This is likely the result of a greater control being needed when swinging at a tempo below the eigenfrequency of the pendulum or their preferred tempo. However, the group differences observed suggest that the obese groups performance is consistently poorer than their normal weight peers regardless of the task difficulty. Surprisingly, we also found gender differences in terms of the amplitude and variability of amplitude. This unexpected and interesting finding is new and rarely found in coordination-based experiments. However, as a gender versus weight group interaction effect was found for CRP, this has led us to tentatively suggest that there may also be differences in the strategies men and women use to coordinate. However, as there is very little evidence demonstrating gender differences in coordination-based literature at present, it potentially could be the result of differences in the proprioception ability or muscle mass between males and females that is influenced by obesity. The coordination of movement (identification of stimulus and coordinated movement patterns in response to stimulus) is a vital part of the successful completion of many activities of daily living in addition to the engagement in physical activity. As such, difficulties in the integration of sensory information to aid coordination could lead to a vicious cycle of inactivity. At present, obese individuals everyday activities are often impeded due to mechanical constraints of excess mass. These visuospatial difficulties demonstrated in this article can be seen as an additional barrier, which obese individuals faced to deal with on a daily basis. The present study adds substantial weight to the hypothesis that the sensory integration process is affected by obesity. However, it is currently unclear how obesity influences this process or whether the difficulties result from problems in the perception, programming, or initiation stage of movement. Future studies may be able to address whether these difficulties are as a result of the differences in the attentional process or gaze strategies employed by normal weight and obese groups through the use of eye tracking software. In addition to this, future studies could ensure subjects swing in the frontal plane with the stimulus presented directly in front of them to eliminate any additional attentional demand placed on subjects as a result of being required to turn their head. In the current study we sought to specifically examine the sensory integration process of a visual motor task while controlling for the mechanical constraints associated with obesity.

The presence of differences between groups suggests variation in both ability and quality movement control mechanism as a result of obesity. This dissimilarity supports the findings of previous studies suggesting the existence of sensory integration deficiencies between obese and normal weight subjects [9, 10, 12, 13, 15]. It remains to be seen if these sensory integration problems exist prior to obesity and can be seen as a contributing factor to becoming obese or whether the development of obesity leads to detrimental consequences for the sensory integration process. In order to answer this question, future research needs to be carried out on children to see if these difficulties emerge over time or whether they exist prior to obesity rather than as a consequence. If these sensory integration difficulties exist prior to and/or as an additional contributing factor to becoming obese, it could allow for the development of targeted interventions to help tackle these difficulties to avoid individuals descending into a downward spiral of physical inactivity as a result of coordination difficulties. Alternatively, this opens the door to questions on whether this sensory integration difficulty is a consequence of the various physiological changes which occur due to obesity that is altered neurotransmitter function, hormonal changes, or nerve signalling [25]. One such hypothesis set out by Scarpina et al. (2016) tentatively suggests that differences in sensory integration between obese and normal weight controls could result from the influence of increased levels of proinflammatory cytokines on the excitation/inhibition balance that regulates neural oscillatory activity. Whatever the source of these differences, the sensory integration of information is a complex phenomenon that emerges as a result of the influence of all the elements within an individual and from the environment around them. As such it is likely that distorted neurotransmitter function, hormonal imbalances, and altered nerve signalling, as a result of increased adiposity, could all contribute to impaired sensory integration process. A final consideration is the influence of subclinical cognitive impairment such as altered executive function, attention, or visuospatial performance. Evidence exists to suggest obesity influences neurocognitive function in adults [24]. Future studies are merited in order to investigate the relationship between obesity, motor control, and neurocognitive functions.

In conclusion, this study suggested visual motor coordination is impaired for obese patients. This finding raises numerous questions in relation to the etiology of these problems, the extent to which these differences influence an individual's life and whether these problems can be remedied or alleviated. Similarly, as we live in a multisensory environment, future studies are also merited to investigate the influence of obesity on multisensory integration process.

## Figures and Tables

**Table 1:** Table of anthropometric measures (mean ± standard deviation) for age, height, weight, and BMI of participants divided by group.

	Age (years)	Height (m)	Weight (kg)	BMI (kg/m^2^)
Normal weight	45.93 ± 10.39	1.71 ± 0.12	64.26 ± 13.22	22.16 ± 1.78
Obese	46.39 ± 10.69	1.72 ± 0.13	153.84 ± 35.19	51.93 ± 8.98
