# Corrigendum to “Impaired Visual Motor Coordination in Obese Adults”

**DOI:** 10.1155/2018/1820890

**Published:** 2018-02-21

**Authors:** David Gaul, Arimin Mat, Donal O'Shea, Johann Issartel

**Affiliations:** ^1^School of Health and Human Performance, Dublin City University, Dublin, Ireland; ^2^Weight Management Service, St. Columcille's Hospital, Loughlinstown, Ireland; ^3^Department of Endocrinology, St. Vincent's University Hospital, Dublin 4, Ireland

In the article titled “Impaired Visual Motor Coordination in Obese Adults,” there was an error in the Consent section, which should be corrected as follows:

“Ethical approval was granted by Dublin City University's research Ethics Committee (DCUREC/2011/038). Prior to commencement of the research study, informed consent from participants was obtained.”
